# Surgical wounding enhances pro‐tumor macrophage responses and accelerates tumor growth and lung metastasis in a triple negative breast cancer mouse model

**DOI:** 10.14814/phy2.15497

**Published:** 2022-11-02

**Authors:** Sierra J. McDonald, Brandon N. VanderVeen, Brooke M. Bullard, Thomas D. Cardaci, Sarah S. Madero, Ioulia Chatzistamou, Daping Fan, E. Angela Murphy

**Affiliations:** ^1^ Department of Pathology, Microbiology & Immunology University of South Carolina School of Medicine Columbia South Carolina USA; ^2^ AcePre, LLC Columbia South Carolina USA; ^3^ Department of Cell Biology and Anatomy University of South Carolina School of Medicine Columbia South Carolina USA

**Keywords:** breast cancer, inflammation, macrophages, metastasis, surgical wounding

## Abstract

Approximately one‐third of all breast cancer mortality results from metastatic recurrence after initial success of surgery and/or therapy. Although primary tumor removal is widely accepted as beneficial, it has long been suspected that surgery itself contributes to accelerated metastatic recurrence. We investigated surgical wounding's impact on tumor progression and lung metastasis in a murine model of triple negative breast cancer (TNBC). Ten‐week‐old female mice were inoculated with 4 T1 cells (week 0) and were either subjected to a 2 cm long cutaneous contralateral incision (wounded) or control (non‐wounded) on week 2 and monitored for 3 weeks (week 5). Mice with surgical wounding displayed significantly accelerated tumor growth observable as early as 1‐week post wounding. This was confirmed by increased tumor volume and tumor weight, post‐mortem. Further, surgical wounding increased metastasis to the lungs, as detected by IVIS imaging, in vivo and ex vivo (week 5). As expected then, wounded mice displayed decreased apoptosis and increased proliferation in both the primary tumor and in the lungs. Flow cytometry revealed that primary tumors from wounded mice exhibited increased tumor associated macrophages and specifically M2‐like macrophages, which are important in promoting tumor development, maintenance, and metastasis. Immunofluorescence staining and gene expression data further confirms an increase in macrophages in both the primary tumor and the lungs of wounded mice. Our data suggests that surgical wounding accelerates tumor progression and lung metastasis in a mouse model of TNBC, which is likely mediated, at least in part by an increase in macrophages.

## Graphical abstract

Our study documents that mice with surgical wounding, at a distant site and without tumor resection, displayed acceleration of primary tumor growth as well as significantly enhanced lung metastasis in a triple negative breast cancer model. Further, wounded mice displayed decreased apoptosis and increased proliferation in both the primary tumor and in the lungs. These responses were associated with an increase in macrophages in both the primary tumor and in the lungs of wounded mice.

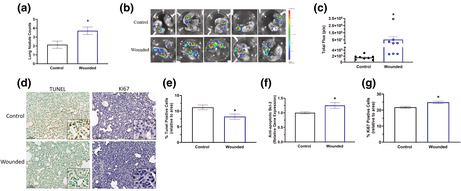

## 1 INTRODUCTION

Despite advances in early detection and treatments, breast cancer remains the most commonly diagnosed cancer and the second leading cause of cancer‐related death in women in the US (Siegel et al., 2022). Although therapies and treatments (e.g. chemotherapy, radiation, surgery) targeting the primary breast tumor have improved (Moo et al., 2018), these strategies remain limited in their efficacy to prevent metastasis (Damen et al., 2021; Waks & Winer, 2019). Indeed, patients with metastatic invasive breast cancer have substantially reduced life quality, efficacious treatment options, and survival rate compared to non‐metastatic invasive breast cancer patients (Waks & Winer, 2019). Given that the 5‐year survival rate for women diagnosed with non‐metastatic localized‐stage breast cancer is 99%, compared to 30% with metastatic distant‐stage breast cancer (American Cancer Society, 2022), there is a clear unmet need to understand the stimuli and associated cellular events that promote tumor cell outgrowth to distant sites.

Primary tumor removal is widely accepted as beneficial (Moo et al., 2018); however, accumulating evidence suggests that the surgical insult itself may trigger early metastatic relapse (Dillekas et al., 2014, 2016; Tohme et al., 2017). Indeed, a staggering 30% of breast cancer patients, regardless of stage and receptor expression status, present with local or distant metastasis following curative surgery (Sauer et al., 2021). Given the short time span of breast cancer patients presenting with early metastatic relapse after surgery and/or treatments (6–24 months) (Cheng et al., 2012; Colleoni et al., 2016), some have postulated that the surgery itself is responsible. Thus, one theory for early metastatic relapse hypothesizes that clinically undetectable cancer cells called micrometastases are present in distant organs prior to surgery but remain dormant and under immune control, until this metastatic dormancy is disrupted by the surgery itself to result in accelerated growth of micrometastases into clinically detectable macrometastases (Demicheli et al., 2007; Dillekas et al., 2014, 2016; Klein, 2020). This is supported by the recent discovery that the dissemination of cancer cells from the primary tumor to distant sites occurs very early in cancer progression (Klein, 2020). While clinical evidence favors this hypothesis, it is difficult to test clinically and experimentally. Using a unique immunogenic, syngeneic breast cancer mouse model, Robert Weinberg's group showed that surgery‐induced tumor outgrowth was associated with the mobilization of myeloid cells into the circulation of wounded mice (Krall et al., 2018). Specifically, they implicated inflammatory monocytes, which differentiate into macrophages in the tumor microenvironment, as likely mediators of the systemic response to surgery (Krall et al., 2018).

Given the dearth of studies examining the link between surgical tumor resection and metastasis, more studies are needed to fully understand the complications behind surgery induced early metastatic breast cancer. Thus, we sought to investigate whether surgery wounding itself, at a distant site and without tumor resection, is sufficient to accelerate primary tumor progression and lung metastasis in a mouse model of triple negative breast cancer (TNBC). We further sought to assess the macrophage response in the tumor following wounding as a plausible mechanism. We used our TNBC cell line, 4 T1‐luciferase2‐red fluorescent protein (modified‐4 T1) which displays delayed breast tumor progression and reduced lung metastatic occurrence in comparison with the rapid growth and metastasis of parental 4 T1 breast tumors (Atiya et al., 2019). Female mice were inoculated with modified‐4 T1 cells (week 0) and were either subjected to a 2 cm long cutaneous contralateral incision (wounded) or control (no wound) 2 weeks later. We reveal that mice with surgical wounding, at a distant site and without tumor resection, displayed acceleration of primary tumor growth and enhanced tumor metastasis in a TNBC model, which may be mediated, at least in part, by the documented increase in macrophage responses. This data adds to the very limited experimental research linking surgical wounding to acceleration of local and distant tumor outgrowth (i.e. metastasis) and suggests a role for pro‐tumor macrophages in this response.

## 2 METHODS

### 2.1 Animals

Five‐week‐old female balb/c (*n* = 20) mice were purchased from Jackson laboratories and cared for in the Department of Laboratory Animal Resources (DLAR) at the University of South Carolina's School of Medicine. Mice were housed 3‐5/cage, maintained on a 12:12‐h light–dark cycle in a low stress environment (22°C, 50% humidity, low noise), and given food and water ad libitum. All mice were fed an AIN‐76A diet (Bioserv; catalog#:F1515), a purified, balanced diet that is phytoestrogen free (Enos et al., 2013; Grotto & Zied, 2010). Dietary phytoestrogens have been shown to influence anxiety‐related behaviors, fat deposition, blood insulin, leptin and thyroid levels as well as lipogenesis and lipolysis in adipocytes, all of which could nonspecifically impact tumorigenesis (Warden & Fisler, 2008). Body weight, food, and water consumption were monitored on a weekly basis for the duration of the study (5 weeks). All experimental mice were euthanized after 5 weeks post breast cancer cell inoculation (15 weeks of age). The Institutional Animal Care and Usage Committee of the University of South Carolina approved all experiments, and all methods were performed in accordance with the American Association for Laboratory Animal Science.

### 2.2 Experimental design

**FIGURE 1:**
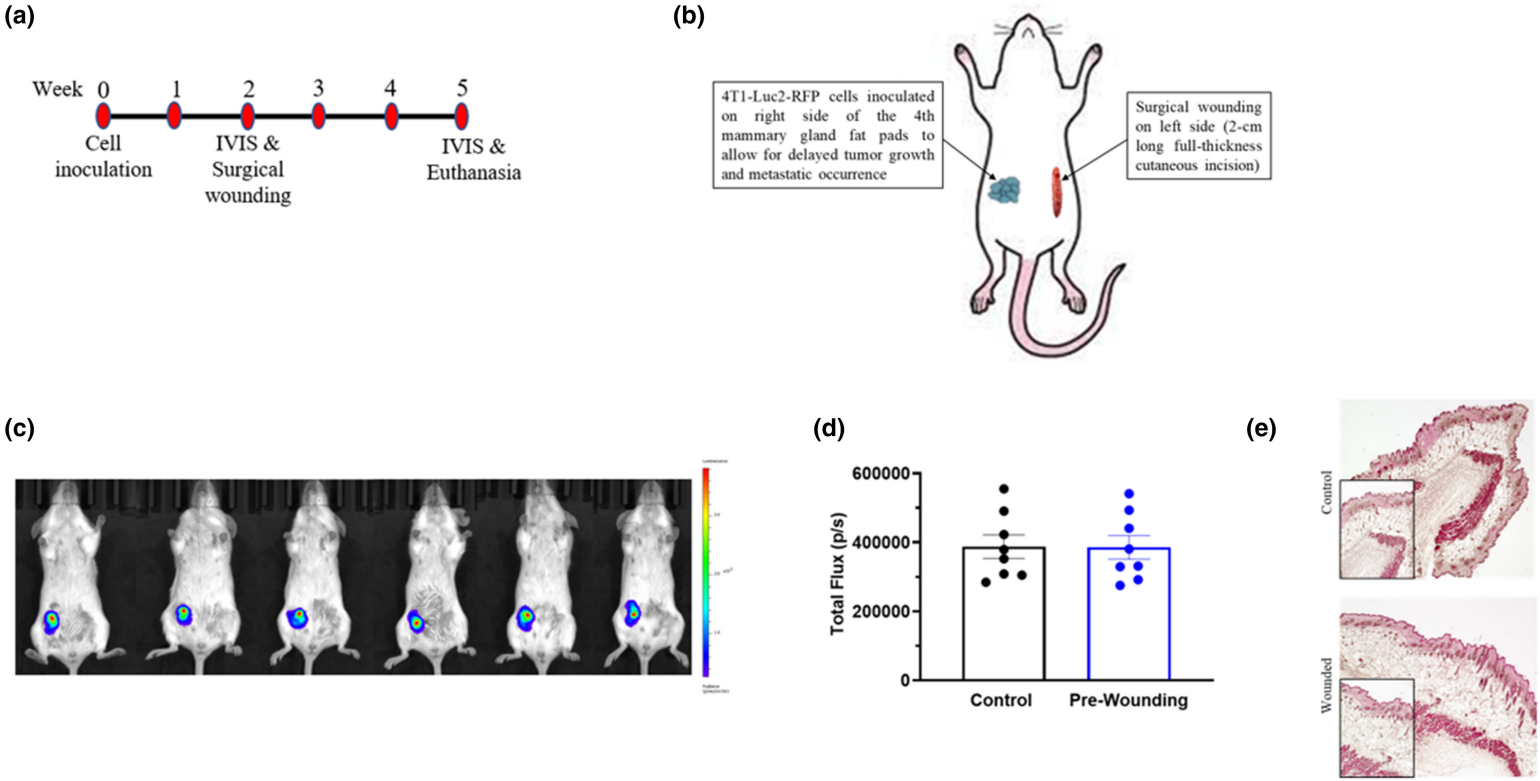
Experimental design and mouse model. (a) Experimental timeline for 10‐week‐old female balb/c mice inoculated with 1 × 10^4^ 4 T1‐Luc2‐RFP cells at week 0. (b) In all mice, 4 T1‐Luc2‐RFP cells were inoculated on right side; mice were either subjected to a 2‐cm long cutaneous incision (wounded) or no incision (control) on the contralateral side of the tumor. (c) Representative bioluminescent images of luciferase carried 4 T1‐Luc2‐RFP tumor cells in all mice before surgical wounding using in vivo IVIS at week 2. (d) Quantification of bioluminescence of luciferase carried 4 T1‐Luc2‐RFP tumor cells represented by the total flux (radiance (photons/sec) in each pixel summed or integrated over the region of interest area. (e) H&E‐stained section of skin on right side of mouse at week 5.

We took advantage of our novel generation of a TNBC cell line, 4 T1‐Luc2‐RFP, that displays delayed breast tumor progression and reduced lung metastatic occurrence in comparison with the rapid growth and metastatic occurrence of parental 4 T1 breast tumors (Atiya et al., 2019). Briefly, 10‐week‐old female balb/c mice (*n* = 20) were inoculated with 1 × 10^4^ 4 T1‐Luc2‐RFP cells on the right side of the 4th pair mammary gland fat pads at week 0 (Figure 1a,b). Once tumors were established, reaching approximately 200 mm^3^ in size 2 weeks post inoculation, mice with similar tumor sizes (determined by IVIS imaging) were randomized to one of two groups (Figure 1c,d), and either subjected to a sterile 2 cm long cutaneous incision (wounded; *n* = 10) or no incision (control; *n* = 10) on contralateral side of the tumor. Thus, a decoupled wounding model was used: an orthotopic tumor inoculated on right side and a surgical wound created on the left side of a mouse. To model surgery wound healing, the incision was allowed to heal (Figure 1e).

### 2.3 Tumor palpations

Tumors were measured beginning at 10 weeks of age (week 0), by the same investigator. Mice develop a palpable mammary tumor 1 week post breast cancer cell inoculation. Upon palpation of a tumor, calipers were used to measure the longest and shortest diameter of the tumor. Tumor volume was estimated using the formula: 0.52 X (largest diameter) X (smallest diameter)^2^ as previously described (Cranford et al., 2019; Steiner et al., 2014).

### 2.4 In vivo measurement of tumor growth and metastasis by IVIS imaging

Bioluminescent imaging was performed using a highly sensitive, IVIS Spectrum in vivo imaging system (PerkinElmer). D‐luciferin (Gold Biotechnology) was prepared per manufacturer's instructions in DPBS and filter sterilized with a 0.2 μm filter for a concentration of 15 mg/ml. For all in vivo imaging, D‐luciferin solution was intraperitoneally injected (150 mg/kg) in all mice for 10 min. Mice were then anesthetized with 2% isoflurane prior and during imaging with IVIS spectrum. Bioluminescence of D‐luciferin carried 4 T1‐Luc2‐RFP tumor cells was quantified using Living Image, in which regions of interest from displayed images were identified and quantified as the total flux which is the radiance (photons/sec) in each pixel summed or integrated over the ROI area.

### 2.5 Tissue collection

Five weeks post breast cancer cell inoculation, mice were euthanized by isoflurane overdose following a 4 h fast. Blood was collected from the inferior vena cava for a blood panel analysis. Breast tumors were excised, weighed (g), and measured (mm^3^) prior to cutting into fourths. One fourth portion of breast tumor was placed in RPMI 1640 medium (ATCC) supplemented with 10% FBS, 1% Penicillin/Streptomycin on ice for flow cytometry. Another one fourth portion of breast tumor was fixed in 4% formaldehyde and paraffin embedded for histopathology, immunohistochemistry, and immunofluorescence. The remaining portions were snap frozen in liquid nitrogen for RT‐PCR gene expression analysis. A portion of the cutaneous skin was then carefully removed on the contralateral side of the tumor where mice were either subjected to a 2 cm incision (wounded) or no incision (control). The skin tissue was fixed in 10% formalin and paraffin embedded for histopathology. Lungs were weighed, imaged on IVIS as described below, and then fixed in 4% formaldehyde and paraffin embedded for histopathology, immunohistochemistry, and immunofluorescence.

### 2.6 Immunohistochemistry

To examine cell proliferation, immunohistochemical analysis of Ki67 (Abcam, #ab16667) was performed according to manufacturer's instructions (abcam IHC staining protocol for paraffin sections) with minor modifications. Formalin fixed paraffin embedded sections (FFPE) (5 μm) were deparaffinized and rehydrated prior to antigen retrieval for 30 min at 95°C with Rodent decloaker (Biocare Medical, #RD913M). Sections were then incubated in 10% goat serum for 2 h at RT in a humid chamber to block nonspecific binding. Sections were labeled with primary antibody against Ki67 (1:100, Abcam, ab16667) overnight at 4°C and then detected with goat anti‐rabbit IgG HRP‐linked antibody (1:100, Cell Signaling, #7074S) for 2 h at RT. Color was developed with DAB (Vector Laboratories, #SK‐4100) as a chromogen for 6 min at RT followed by counterstaining using CAT hematoxylin and Tacha's bluing for 30 secs. Sections were dehydrated and mounted with Permount medium (Fisher Chemical, #SP15‐100). Representative images were taken at ×20 and ×40 using Keyence All‐in‐One fluorescence Microscope BZ‐X800. Percentage of Ki67 cells (DAB‐positive nuclei) over total cells, relative to area, were quantified using Material Image Processing and Automated Reconstruction (MIPAR) software, more specifically, we used MIPAR's immunohistochemistry recipe which quantifies positively stained DAB cells relative to area (Rizzardi et al., 2012). Five images were assessed for each mouse (*n* = 3 mice/group).

TUNEL staining was carried out as per the manufacturer's instructions using ApopTag Peroxidase In Situ Apoptosis Detection Kit (Millipore, #S7100). Color was developed with DAB (Vector Laboratories, #SK‐4100) as a chromogen for 6 min at RT followed by counterstaining using 1% methyl green (R&D systems, #4800‐30‐18) for 10 min at RT and then washed with diH20 and 100% N‐Butanol (Acros‐Organic, #71–36‐3). Sections were dehydrated and mounted with Permount medium (Fisher Chemical, #SP15‐100). Representative images were taken at ×20 and ×40 using Keyence All‐in‐One fluorescence Microscope BZ‐X800. Percentage of apoptotic cells (TUNEL‐positive nuclei) over total cells, relative to area, were quantified using Material Image Processing and Automated Reconstruction (MIPAR) software, more specifically, we used MIPAR's immunohistochemistry recipe which quantifies positively stained DAB cells relative to area (Rizzardi et al., 2012). Five images were assessed for each mouse (*n* = 3 mice/group).

### 2.7 Immunofluorescence

After deparaffinization and rehydration, paraffin sections were incubated with rodent decloaker (Biocare Medical, #RD913M) for 30 min at 95°C for antigen retrieval and then washed with TBS. To block nonspecific binding, sections were incubated with 10% goat serum in 5% BSA/TBS for 1 h at RT in a humid chamber under gentle agitation. Following the removal of the block buffer, sections were labeled with primary antibody against F4/80 (1:100, Santa Cruz, #sc‐377009) overnight at 4°C, washed with TBS, and then detected with Alexa Fluor 488 goat anti‐rat secondary antibody (1:200, Abcam, ab150157) in 5% BSA/TBS for 30 min at RT in a humid chamber under gentle agitation in the dark. Sections were then washed with TBS and counterstained with 4′,6‐diamidino‐2‐phenylindole (DAPI, 1ug/ml). Sections were washed with water and mounted with a glycerol based mounting media. Representative fluorescent images were taken at ×20 using Leica DM2500 fluorescent microscope.

### 2.8 Blood panel analysis

A complete blood panel analysis was performed using the VetScan HMT (Abaxis) for determination of white blood cells (WBC), lymphocytes (LYM), monocytes (MON), neutrophils (NEU), red blood cells (RBC), hematocrit (Hct), and hemoglobin (Hb). The neutrophil to lymphocyte ratio (NLR) was calculated from obtained values. Briefly, whole blood was placed in an EDTA coated microtube and analyzed on the VetScan HMT according to the manufacturer's instructions.

### 2.9 Ex vivo measurement of tumor growth and metastasis by IVIS imaging

For ex vivo imaging, lungs were carefully washed in a well containing PBS, and then transferred to another well in which D‐luciferin (300 μg/ml) was added to cover the tissue. Ex vivo lungs were immediately imaged on IVIS to determine lung nodule counts. Bioluminescence of D‐luciferin carried 4 T1‐Luc2‐RFP tumor cells was quantified as described above.

### 2.10 Flow cytometry

One fourth portion of breast tumors were placed on ice in 5 ml RPMI 1640 medium (ATCC) supplemented with 10% FBS, 1% Penicillin/Streptomycin. Each tumor was cut into small fragments with a blade and enzymatically digested in 1 ml of RPMI 1640 medium (ATCC) supplemented with 10% FBS, 1% Penicillin/Streptomycin, 0.3 mg/ml of collagenase Type 4 (Worthington), 200 U/ml of DNase I (Worthington), and 1 U/mL of Hyaluronidase (Sigma‐Aldrich) for 60 min at 37°C. After digestion, cell suspensions were passed through 70‐μm cell strainers. Cells were then washed with PBS, passed through 70‐μm cell strainers, and washed with PBS. For the staining of cell surface markers (Biolegend unless otherwise indicated), appropriate samples were stained with ZombieGreen (FITC; Bio‐Rad) solution for 20 min at RT in dark and then washed with flow buffer (0.5% FBS, 10 mM HEPES (Gibco), and 2 mM EDTA (Gibco) in HBSS without calcium or magnesium). Cells were incubated with FC Block solution for 10 min at 4°C and then incubated with fluorescently labeled antibodies against CD45 (PE/Cy7), CD11b (APC), CD68 (APC/Cy7), CD206 (PE), and CD11c (PerCP/Cy5.5) for 20 min at 4°C, followed by two PBS washes with the final resuspension in flow buffer. Cells were measured using a FACS Aria II (BD) and analyzed using FlowJo (BD Biosciences).

Breast tumors cells were gated for non‐debris singlets and considered live immune cells with ZombieGreen^Neg/Low^ and CD45^+^. From the Live CD45^+^ population, CD11b^+^CD68^+^ cells were identified as macrophages. From the CD11b^+^CD68^+^ macrophage population, macrophage phenotype was determined based on CD206 and CD11c expression. CD206^−^CD11c^−^ cells were identified as M0 macrophages; CD206^−^CD11c^+^ as M1 macrophages; CD206^+^CD11c^−^ cells as M2 macrophages; CD206^+^CD11c^+^ as M1‐M2 transitional macrophages.

### 2.11 Real‐time quantitative PCR

Mammary tumors and lungs were homogenized, and RNA was extracted using the RNeasy Lipid Tissue Mini Kit (Qiagen, Cat#74804) according to the manufacturer's instructions. RNA sample quality and quantities were verified using a Nanodrop One Microvolume UV–Vis Spectrophotometer (Thermo Fisher Scientific, Waltham, MA, United States) and determined to be of good quality based on A260/A280 and 260/230 values (>1.8) prior to cDNA synthesis using High‐capacity Reverse Transcriptase kit (Applied Biosystems, Cat# 4368814). Quantitative reverse transcriptase polymerase chain reaction (PCR) analysis was carried out as per the manufacturer's instructions and all primers used were TaqMan Gene Expression Assays (Applied Biosystems). TaqMan reverse transcription reagents were used to reverse transcribe and to analyze mammary tumors mRNA gene expression of the following markers: monocyte/macrophage (EMR1/F480 and CD68), pro‐inflammatory M1 macrophage markers (IL‐6 and TNF‐a), anti‐inflammatory M2 macrophage markers (IL‐4, IL‐10, IL‐13 and TGF‐β1), neutrophils (Ly6G), T cell subsets (Foxp3, CD4, and CD8), chemokines (CCL2 and CCL5) and angiogenesis (metalloproteinase‐9 [MMP9]; lungs only) was performed as previously described (Enos et al., 2015). Briefly, quantitative RT‐PCR analysis was carried out as per the manufacturer's instructions (Applied Biosystems) using Taq‐Man Gene Expression Assays on a Qiagen Rotor‐Gene Q. Data were normalized to controls (non‐wounded mice) and compared to five reference targets (Hmbs, B2M, TBP, H2afv, and 18 s), which were evaluated for expression stability using GeNorm.

### 2.12 Histopathology analysis

A portion of skin tissue contralateral to tumor from each mouse was fixed overnight in 10% formalin, dehydrated with alcohol, and embedded in paraffin wax. Paraffin sections were stained with hematoxylin and eosin (H&E) (Fisher HealthCare; catalog#: 245–651 and 245–827) as previously described by our group (Cranford et al., 2019). Histological analyses were performed blindly by a certified pathologist (I.C).

### 2.13 Statistical analyses

All data were analyzed using commercial software (GraphPad Software, Prism 7). All in vivo outcomes were analyzed using a student's *t*‐test or one‐way ANOVA where appropriate. The strength of the linear relationship between select outcomes was assessed using a Pearson correlation. Statistical significance was set with an alpha value of *p* ≤ 0.05. Data are presented as mean ± standard error of mean (SEM). All figures, excluding IVIS, were generated using GraphPad Prism.

## 3 RESULTS

### 3.1 Experimental design and animal characteristics

The experimental design is presented in Figure 1a. There were no differences in body weight detected across the experimental period (data not shown). Two weeks post inoculation, in vivo IVIS imaging was performed to visualize the tumors; it was confirmed that no differences in tumor growth were detected before surgery wounding (Figure 1c). Mice were evenly grouped based on the bioluminescence of luciferase carried 4 T1‐Luc2‐RFP tumor cells, which is represented by the total flux (radiance [photons/sec] in each pixel summed or integrated over the region of interest area; Figure 1d). There were no signs of infection at the wound (monitored daily) in any mice over the study duration. At study completion, the wound was assessed by a pathologist (I.C.); there were no indications of infection in either group and no observable differences in the healing process (Figure 1e).

### 3.2 Surgery wounding accelerates breast tumor progression

**FIGURE 2:**
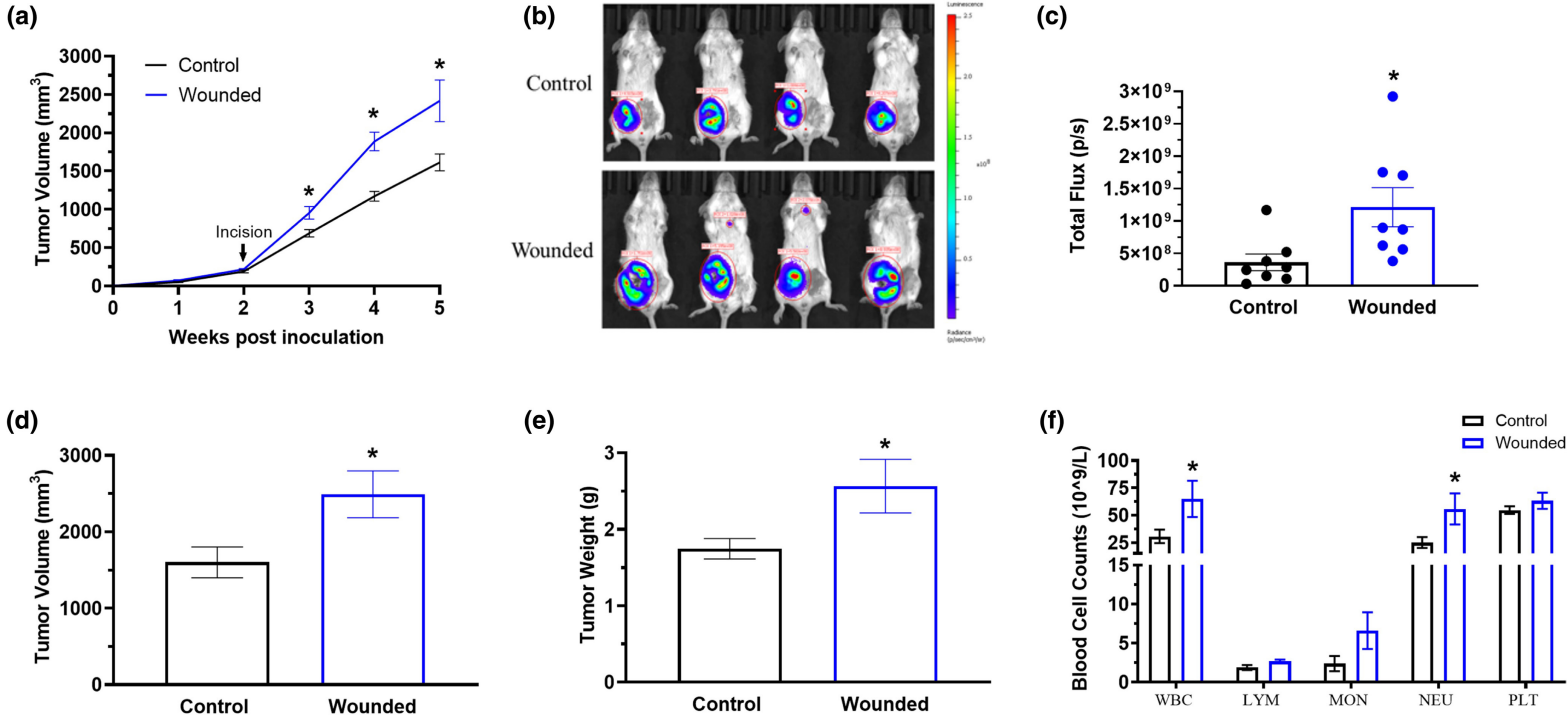
Surgery wounding accelerates breast tumor progression. Control and wounded orthotopic breast tumor bearing female balb/c mice (*n* = 16) were euthanized 5 weeks post 4 T1‐Luc2‐RFP cell inoculation. (a) Weekly tumor volume palpations given in millimeters^3^ (mm^3^). (b) Representative bioluminescent images of luciferase carried 4 T1‐Luc2‐RFP tumor cells using in vivo IVIS before euthanasia. (c) Quantification of bioluminescence of luciferase carried 4 T1‐Luc2‐RFP tumor cells represented by the total flux (radiance (photons/sec) in each pixel summed or integrated over the region of interest area. Tumors excised from control and wounded mice for (d) tumor volume given in millimeters^3^ (mm^3^) and (e) tumor weight in milligrams (mg). (f) Circulating white blood cells (WBCs), lymphocytes (LYM), monocytes (MON), neutrophils (NEU), and platelets (PLT) determined in whole blood using VetScan HM5. Significance was set at *p* < 0.05. *Significantly different from control using a student's *t*‐test.

Mice with surgical wounding displayed significant accelerated tumor growth via greater tumor volume palpitations at weeks 3–5 post breast cancer cell inoculation (Figure 2a; *p* < 0.05). This was confirmed by increased total flux on IVIS in vivo (Day 38; Figure 2b,c; *p* < 0.05). Further, at euthanasia, wounded mice exhibited increased tumor volume (Figure 2d; *p* < 0.05) and tumor weight (Figure 2e; *p* < 0.05) compared to control. No significant differences were observed for all measured organ weights (data not shown).

### 3.3 Surgery wounding increases circulating white blood cells

Significantly elevated circulating white blood cells (WBCs) (*p* < 0.05) and neutrophils (*p* < 0.05) both indicative of systemic inflammation, were exhibited in surgery wounded mice (Figure 2f). An increase in monocytes also was observed with surgical wounding, but this did not reach statistical significance (Figure 2f). The neutrophil to lymphocyte ratio (NLR) was calculated given its association with growth and migration of malignant cells and was found to be increased in wounded mice (13.21 ± 2.24 in control versus 19.98 ± 6.70 in wounded; *p* < 0.05).

### 3.4 Surgery wounding enhances primary breast tumor cell proliferation and reduces apoptosis

**FIGURE 3:**
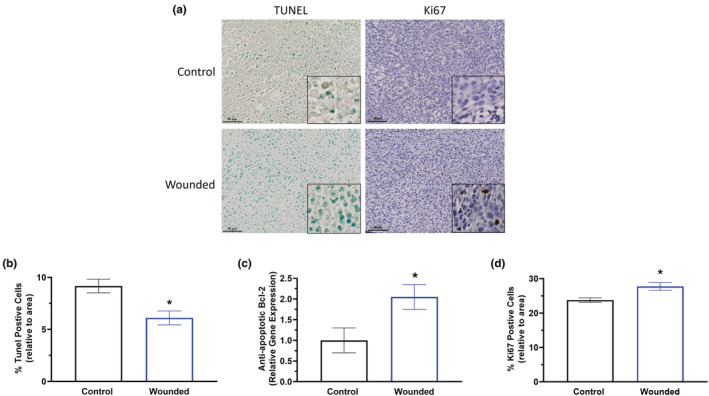
Surgery wounding decreases apoptosis and increases proliferation in the primary tumor. Immunohistochemistry of breast tumor sections from control and wounded mice (*n* = 3/group) stained with TUNEL for detection of apoptotic cells and Ki67 to examine proliferation. (a) Representative 20× images with 40× insets of breast tumors stained with TUNEL (left) and Ki67 (right). Scale bar = 50 μm. (b) Percentage of apoptotic (positively stained TUNEL‐DAB) cells relative to area. (c) Gene expression analysis of Bcl‐2, an anti‐apoptotic marker, within the tumor using qPCR. (d) Percentage of Ki67 positive cells (positively stained Ki67‐DAB) cells relative to area. Significance was set at *p* < 0.05. *Significantly different from control using a student's *t*‐test.

To further investigate the pro‐tumoral effects associated with surgical wounding, breast tumor sections were stained with TUNEL for detection of apoptotic cells (Figure 3a left panels) and Ki67 to examine proliferation (Figure 3a right panels). Surgical wounding significantly decreased the percentage of apoptotic (positively stained) cells (Figure 3a,b; *p* < 0.05) (i.e. the number of TUNEL positive cells over total cell number relative to area). Additionally, gene expression of anti‐apoptotic marker, Bcl‐2 also was significantly increased in breast tumors of surgical wounded mice compared to control (Figure 3c; *p* < 0.05). Further, surgical wounding resulted in a significant increase in the percentage of Ki67 positive cells (*p* < 0.05) in breast tumors compared to control (Figure 3a,d).

### 3.5 Surgery wounding increases lung metastasis in association with increased proliferation and decreased apoptosis

**FIGURE 4:**
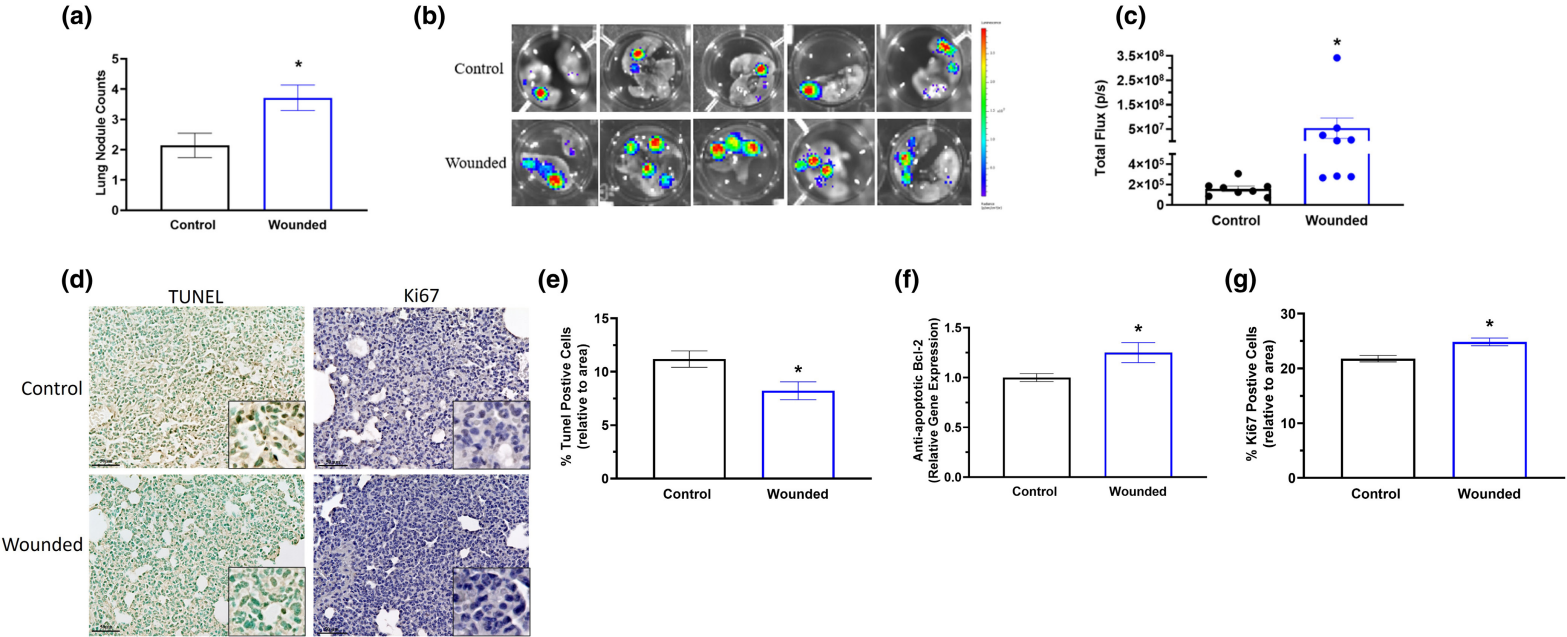
Surgery wounding increases lung metastasis. Control and wounded orthotopic breast tumor bearing female balb/c mice (*n* = 16) were euthanized 5 weeks post 4 T1‐Luc2‐RFP cell inoculation. (a) Lung nodules were determined and counted from IVIS images of ex vivo lungs. (b) Representative bioluminescent images of luciferase carried 4 T1‐Luc2‐RFP tumor cells in ex vivo lungs using IVIS immediately after euthanasia. (c) Quantification of bioluminescence of luciferase carried 4 T1‐Luc2‐RFP tumor cells in ex vivo lungs represented by the total flux (radiance (photons/sec) in each pixel summed or integrated over the region of interest area. Immunohistochemistry of lung sections from control and wounded mice (n = 3/group) stained with TUNEL for detection of apoptotic cells and Ki67 to examine proliferation. (d) Representative 20x images with 40x insets of lungs stained with TUNEL (left) and Ki67 (right). Scale bar = 50 μm. (e) Percentage of apoptotic (positively stained TUNEL‐DAB) cells relative to area. (f) Gene expression analysis of Bcl‐2, an anti‐apoptotic marker, within the lung using qPCR. (g) Percentage of Ki67 positive cells (positively stained Ki67‐DAB) cells relative to area. Significance was set at *p* < 0.05. *Significantly different from control using a student's *t*‐test.

Significantly increased number of nodules on ex vivo lungs was exhibited in surgical wounded mice (*p* < 0.05; Figure 4a). To further visualize and measure metastasis, IVIS was performed to quantify the total flux (bioluminescence) of luciferase carried 4 T1‐Luc2‐RFP tumor cells. Surgical wounding significantly increased metastasis as indicated by increased total flux for IVIS both in vivo lungs (Figure 2b,c; *p* < 0.05) and ex vivo lungs (Figure 4b,c; *p* < 0.05). Consistent with the primary tumor data, surgical wounding significantly decreased the percentage of apoptotic (positively stained) cells in the lungs (Figure 4d,e; *p* < 0.05) (i.e. the number of TUNEL positive cells over total cell number relative to area). Additionally, gene expression of anti‐apoptotic marker, Bcl‐2 was significantly increased in the lungs of surgical wounded mice compared to control (Figure 4f; *p* < 0.05). Further, surgical wounding resulted in a significant increase in the percentage of Ki67 positive cells (*p* < 0.05) in the lungs compared to control (Figure 4d,g).

### 3.6 Surgery wounding increases macrophages and associated mediators in the primary tumor

**FIGURE 5:**
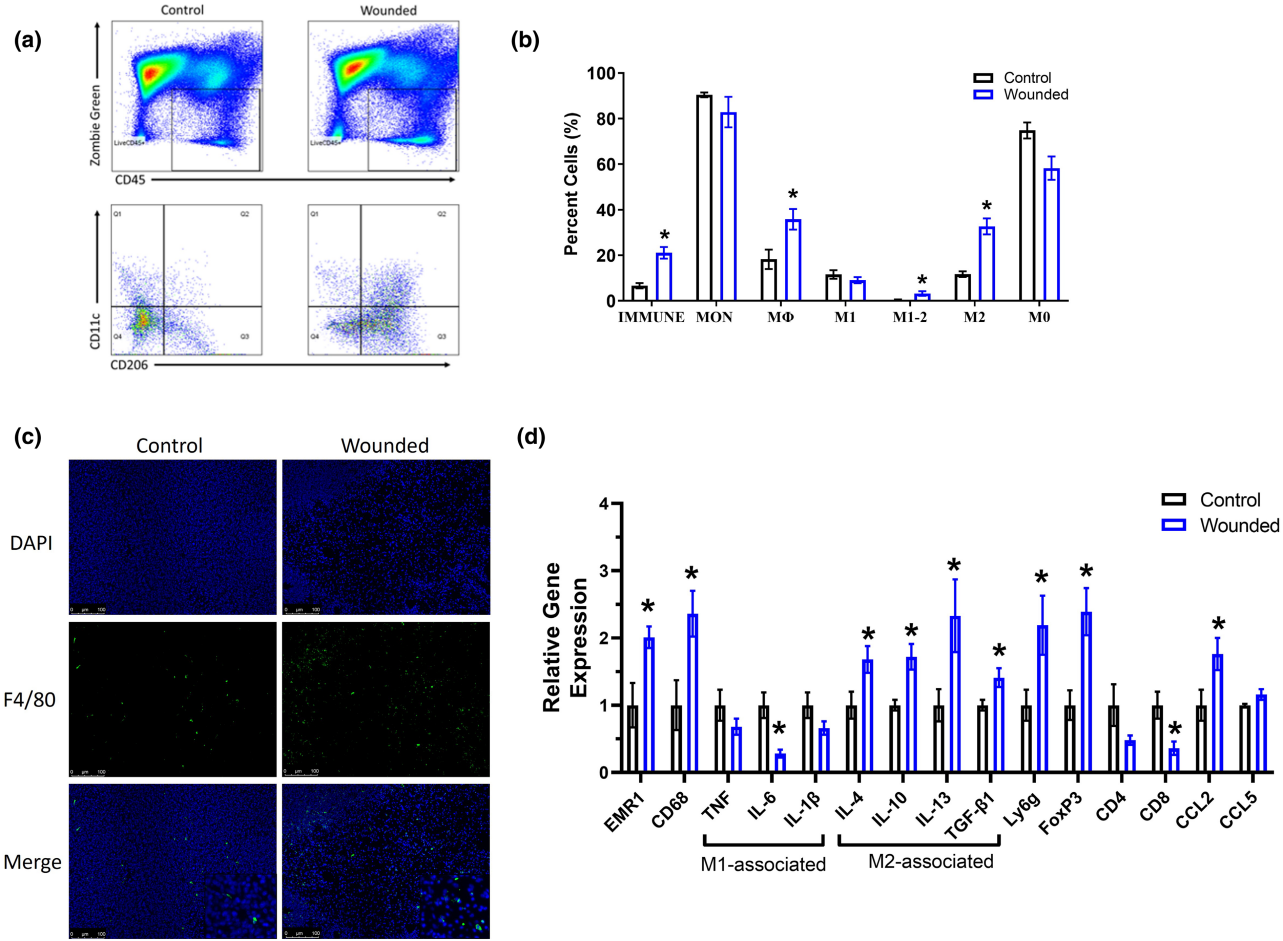
Surgery wounding upregulates pro‐tumor M2 macrophages in the primary tumor. Control and wounded orthotopic breast tumor bearing female balb/c mice (*n* = 16) were euthanized 5 weeks post 4 T1‐Luc2‐RFP cell inoculation. Breast tumors cells were gated for non‐debris singlets and considered live immune cells with ZombieGreen^Neg/Low^ and CD45^+^. From the Live CD45^+^ population, CD11b^+^CD68^+^ cells were identified as macrophages. From the CD11b^+^CD68^+^ macrophage population, CD206^−^CD11c^−^ cells were identified as M0 macrophages; CD206^−^CD11c^+^ as M1 macrophages; CD206^+^CD11c^−^ cells as M2 macrophages; CD206^+^CD11c^+^ as M1‐M2 transitional macrophages. (a) Representative flow plots identifying live immune cells (CD45^+^), and M0 (CD206^−^CD11c^−^), M1 (CD206^−^CD11c^+^), M2 (CD206^+^CD11c^−^ cells), M1‐M2 transitional (CD206^+^CD11c^+^) macrophages within breast tumors. (b) Percentages of immune cells, monocytes (MON), macrophages, and M1, transitional M1‐M2, M2, and M0 macrophages. (c) Representative 20x images of immunofluorescence staining of F4/80 in breast tumor tissues harvested from control and wounded mice. DAPI (blue) as an individual channel (top) and F4/80 (green) as an individual channel (middle) for visualization of nuclei, and merged (bottom). Scale bar = 100 μm. (d) qPCR analysis of the following markers: monocyte/macrophage (EMR1/F480 and CD68), pro‐inflammatory M1 macrophage markers (TNF‐a, IL‐6, and IL‐1β), anti‐inflammatory M2 macrophage markers (IL‐4, IL‐10, IL‐13 and TGF‐β1), neutrophils (Ly6G), T cells (regulatory T cells [Foxp3], CD4 and CD8), and chemokines (CCL2 and CCL5). Data were normalized to controls and compared with five reference targets (B2M, TBP, HPRT, HMBS, and H2AFV), which were evaluated for expression stability using GeNorm. Significance was set at *p* < 0.05. *Significantly different from control using a student's *t*‐test.

To investigate the pro‐tumoral effects associated with surgical wounding, breast tumors were analyzed to examine macrophage markers by flow cytometry. Representative flow plots of live immune cells (CD45^+^), and M0 (CD206^−^CD11c^−^), M1 (CD206^−^CD11c^+^), M2 (CD206^+^CD11c^−^ cells), M1‐M2 transitional (CD206^+^CD11c^+^) macrophages within breast tumors (Figure 5a). We demonstrate that surgical wounding significantly increased the expression of immune cells (CD45^+^) (*p* < 0.05), macrophages (CD68^+^) (*p* < 0.05), transitional M1‐M2 macrophages (CD206^+^CD11c^+^) (*p* < 0.05) and M2 macrophages (CD206^+^) (*p* < 0.05), important in contributing to tumor growth and immunosuppressive function. Surgical wounding also resulted in a trending reduction in M0 (CD206^−^CD11c^−^) (*p* = 0.057) and a slight decrease in M1 (CD11c^+^) macrophage expression (Figure 5b) but these did not reach statistical significance. The reduction in macrophages exhibited by surgical wounding was confirmed with immunofluorescence staining for F4/80 in the breast tumor (Figure 5c).

To confirm macrophage regulation pro‐tumoral effects associated with surgical wounding, breast tumors were analyzed to examine mRNA expression of macrophage markers via RT‐PCR. Consistent with the flow cytometry and immunofluorescence data, tumors of wounded mice exhibited significantly increased total macrophages as indicated by increased expression of EMR1 and CD68 (*p* < 0.05; Figure 5d), and significantly upregulated the expression of M2 related macrophage markers, including IL‐4, IL‐10, IL‐13, and TGF‐β (*p* < 0.05) (Figure 5d). Further, tumors of wounded mice exhibited significantly reduced anti‐tumoral M1 macrophage cytokine expression of IL‐6 (*p* < 0.05) and a slight but non‐significant reduction in the expression of TNF‐α and IL‐1β (Figure 5d). In addition, surgery wounding also significantly supported an increase in neutrophils and regulatory T cells in the primary tumor, indicated by increased expression of lymphocyte antigen 6 complex locus G6D (Ly6G, *p* < 0.05) and forkhead box P3 (Foxp3, *p* < 0.05; Figure 5d), respectively. Whereas a decrease in the expression of CD8 (*p* < 0.05), indicative of a reduction in cytotoxic T cells, was noted in wounded mice (Figure 5d). Finally, we document an increase in chemokine CCL2 (*p* < 0.05), the most important chemoattractant for macrophages, in wounded mice there was no chance in CCL5 (Figure 5d).

### 3.7 Surgery wounding increases macrophages in the lungs and upregulates mRNA expression of select macrophage markers

**FIGURE 6:**
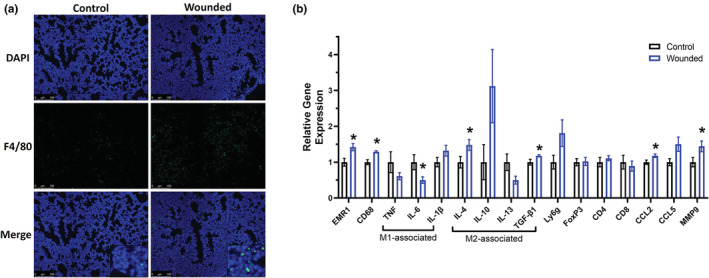
Surgery wounding increases F4/80 in the lungs and upregulates mRNA expression of select pro‐tumor markers. Control and wounded orthotopic breast tumor bearing female balb/c mice (*n* = 16) were euthanized 5 weeks post 4 T1‐Luc2‐RFP cell inoculation. Immunofluorescence staining and qPCR were carried out to assess macrophages and pro–tumor mediators in the lungs. (a) Representative 20× images of immunofluorescence staining of F4/80 in lung tissues harvested from control and wounded mice. DAPI (blue) as an individual channel (top) and F4/80 (green) as an individual channel (middle) for visualization of nuclei, and merged (bottom). Scale bar = 100 μm. (b) qPCR analysis of the following markers in lung tissues: monocyte/macrophage (EMR1/F480 and CD68), pro‐inflammatory M1 macrophage markers (TNF‐a, IL‐6, and IL‐1β), anti‐inflammatory M2 macrophage markers (IL‐4, IL‐10, IL‐13 and TGF‐β1), neutrophils (Ly6G), T cells (regulatory T cells [Foxp3], CD4 and CD8), chemokines (CCL2 and CCL5), and angiogenesis marker (metalloproteinase‐9 [MMP9]). Data were normalized to controls and compared with five reference targets (B2M, TBP, HPRT, HMBS, and H2AFV), which were evaluated for expression stability using GeNorm. Significance was set at *p* < 0.05. *Significantly different from control using a student's *t*‐test.

Immunofluorescence staining for F4/80 in the lungs revealed an increase in macrophages (Figure 6a). Similar to the primary tumor, we analyzed mRNA expression of macrophage related markers via RT‐PCR in the lungs to provide insight into their role in surgical wounding‐induced metastasis. Consistent with the immunofluorescence data, lungs of wounded mice exhibited significantly increased total macrophages as indicated by increased expression of EMR1 and CD68 (*p* < 0.05; Figure 6b), and significantly upregulated expression of select M2 related macrophage markers, including IL‐4 and TGF‐β (*p* < 0.05; Figure 6b). Further and consistent with the primary tumor, lungs of wounded mice revealed significantly reduced anti‐tumoral M1 macrophage cytokine expression of IL‐6 (*p* < 0.05; Figure 6b). However, there were no statistically significant changes in neutrophil or T cell markers in the lungs (Figure 6b). Like in the primary tumor, CCL2 was increased in wounded mice (*p* < 0.05) and there was a trend for an increase in CCL5 (*p* = 0.06; Figure 6b). Finally, the angiogenesis marker MMP9 (*p* < 0.05) was increased with wounding (Figure 6b).

### 3.8 Positive correlations between primary tumor, lung metastasis, and macrophages

In order to further address the relationship between the primary tumor and metastasis and between tumorigenesis outcomes (primary tumor and metastasis) and macrophages we performed correlation analyses (Table 1). There was a positive correlation between primary tumor volume and lung metastasis (i.e. # of nodules) (*R* = 0.60; *p* < 0.05) and between primary tumor flux and secondary tumor (i.e. metastasis) flux (*R* = 0.65; *p* < 0.05). Further, we found a positive correlation between primary tumor macrophage number and both primary tumor volume (*R* = 0.62; *p* < 0.05) and lung metastasis (i.e. # of nodules; *R* = 0.83; *p* < 0.05). Similarly, we note a positive correlation between primary tumor M2 macrophage number and primary tumor volume (*R* = 0.59; *p* < 0.05), primary tumor weight (*R* = 0.59; *p* < 0.05), and lung metastasis (i.e. # of nodules; *R* = 0.58; *p* < 0.05).

**TABLE 1:** Correlations

	Primary Tumor Volume (mm3)	Primary Tumor Weight (g)	Primary Tumor Flux (p/s)	Lung Metastases (# nodules)
Lung Metastases (# nodules)	0.60*	0.47	—	—
2̊ Tumor Flux (p/s)	—	—	0.65*	—
1̊ Tumor Macrophages (# Live CD45+ Cells)	0.62*	0.51	—	0.83*
2̊ Tumor M2‐like Macrophages (% CD11b + CD68+ Cells)	0.59*	0.59*	—	0.58*

*Note*: Pearson's correlation (R).

* Statistically significant correlation (*p* < 0.05).

## 4 DISCUSSION

It has been postulated that surgery and the subsequent wound healing response play a role in the acceleration of tumor metastasis (Cole et al., 2001; Gianni et al., 2011); however, data on the complications surrounding surgery induced early metastatic breast cancer are limited. We demonstrate that surgery wounding itself, on the contralateral side of the tumor, is sufficient to accelerate both tumor growth and lung metastasis in a 4 T1 orthotopic model of TNBC. Indeed, surgical wounded mice exhibited significantly increased primary tumor growth, as well as in vivo and ex vivo lung metastasis compared to non‐wounded mice. These findings were consistent with a decrease in apoptosis and an increase in proliferation in wounded mice. Further, the increase in tumorigenesis and metastasis in surgical wounded mice was associated with promotion of pro‐tumor macrophage responses in both the primary tumor and in the lungs.

Metastasis, accounting for approximately 67%–90% of all cancer mortality, can occur months or years after initial success of surgery and/or therapies (Dillekas et al., 2019; Riggio et al., 2021; Sauer et al., 2021). The reason behind certain cancers and their subtypes relapsing within the first year versus several years after surgery and/or treatments remains largely unknown. It has only recently been established that the dissemination of cancer cells from the primary tumor to distant sites occurs very early in cancer progression (Klein, 2020). Indeed, it has been proposed that clinically undetectable, early disseminated cancer cells (DCCs) are present at distant organs prior to treatment and/or surgery, but remain dormant and under immune control, until this metastatic dormancy is disrupted to result in accelerated growth of DCCs (i.e. micrometastases) into clinically detectable macrometastases. However, the triggering event required to break this metastatic dormancy remains unclear. Chemotherapy‐induced inflammation but more plausibly, the surgery itself, have been suggested to disrupt metastatic dormancy thus awakening DCCs from cell cycle arrest to result in early metastatic relapse (Klein, 2020). Our current findings confirm the surgery hypothesis and aligns with Weinberg's work (Krall et al., 2018), albeit in a different model. Indeed, in the current study we show that surgery wounding itself, without tumor resection, is sufficient to accelerate tumor progression and lung metastasis in a TNBC model. Further support for this comes from the documented decrease in apoptosis and the increase in proliferation in both the primary tumor and the lungs of wounded mice. The relationship between primary tumor volume and lung metastasis is also worth noting; indeed, a positive and significant correlation was documented between these two outcomes lending further support for the hypothesis that surgical wounding promotes acceleration of the primary tumor in concert with enhanced distant tumor outgrowth (i.e. metastasis).

Given that peripheral blood cells play a vital role in the diagnosis and treatment of breast cancer patients (Chen et al., 2020), we examined the impact of wounding on circulating blood cells. In our study, increased metastasis in surgical wounded mice was associated with elevated WBCs, which is consistent with a recent study reporting an association between increased WBCs, enhanced tumor growth, and lung metastasis in 4 T1 bearing mice (Wang et al., 2015). Elevated neutrophils and NLR have been consistently correlated to poor prognosis, lymph node and overall metastasis, and shorter disease‐free and overall survival time in breast cancer patients (Chen et al., 2020; Orditura et al., 2016) and we show that surgical wounding increased neutrophils and NLR. Similarly, increased monocytes have been indicative of increased tumor cell growth and angiogenesis given their ability to produce inflammatory mediators such as vascular endothelial growth factor (VEGF) and TNF‐α (Chen et al., 2020). Together, our data suggests that surgical wounding promotes systemic inflammation that may not only aid in wound healing, but also aid in the further recruitment of these increased circulating WBCs, neutrophils, monocytes, and platelets to both the primary tumor site and to the micrometastases in distant organs (lungs) to facilitate primary and distant tumor growth. However, whether this increase in systemic inflammation is a direct consequence of the wounding itself, a reflection of increased tumor growth, or both, could not be determined from the current study. Given that the wound was healed at the time of assessment of systemic inflammation, we can speculate that the increase is largely due to the increased tumor growth; additional studies that examine systemic inflammation over time following wounding are necessary to draw firm conclusions on the stimulus driving this response.

The process of surgical wound healing and metastasis share similarities in their recruitment of immune cells, including macrophages. Within the breast tumor microenvironment, macrophages facilitate tumor development and metastasis through promoting angiogenesis, tumor cell proliferation, and tumor invasiveness (Medrek et al., 2012; Qian & Pollard, 2010; Rudnick & Kuperwasser, 2012). Macrophages also carry out the same functions of stimulating angiogenesis, host defenses, cell proliferation, and tissue restoration in wound healing (Ceelen et al., 2014). Thus, it is plausible that surgical wounding creates a favorable environment of newly attracted host cells, most notably macrophages, with the capability of stimulating distant or local tumor growth. Indeed, it has been previously reported that inflammatory monocytes, precursors of macrophages, are mobilized into circulation following surgical wounding, increasing their availability for recruitment into tumors and suggesting a mechanism whereby surgical wounding may promote metastasis (Krall et al., 2018). In support of this, we demonstrate via flow cytometry that surgical wounding does in fact increase macrophages in the primary tumor, and specifically M2‐like macrophages. Immunofluorescence staining for F4/80, a pan macrophage marker, confirms this finding. It is also worth noting that this increase in macrophages also was reflected in the lungs. Further, support for a potential causal relationship between surgical wounding accelerated tumor growth and metastasis and pro‐tumor macrophages comes from correlational analysis; we show a significantly positive relationship between M2 macrophages in the primary tumor and (1) primary tumor volume, (2) primary tumor weight, and (3) number of lung nodules.

The cellular data was further confirmed by our gene expression data; primary breast tumors and lungs from surgical wounded mice had upregulated mRNA gene expression of select macrophage markers, and specifically M2 macrophage markers. M2 related markers are essential regulators of the tumor microenvironment and have been implicated in promoting cancer cell evasion, survival, proliferation, and metastasis (Kwasniak et al., 2019; Mirlekar, 2022). Indeed, studies have documented that targeting M2 markers (e.g. IL‐4 and/or IL‐13) can reduce primary breast tumor burden and prevent breast cancer metastasis (Little et al., 2019; Papageorgis et al., 2015; Park et al., 2017; Venmar et al., 2014). Interestingly, expression of CCL2, perhaps more commonly known as MCP‐1, the most important chemokine for macrophage recruitment was increased in both the primary tumor and the lungs providing further support for the relationship between macrophages and tumor growth and metastasis in this model. Further, in support of our findings showing surgery wounding upregulates expression of neutrophils in the primary tumor and in the lungs (trend of p = 0.058), Ly6g + neutrophils were reported to be the main component and driver of lung metastatic establishment in mouse breast cancer models (Wculek & Malanchi, 2015). Similarly, induction of FoxP3+ Tregs also has been shown to promote 4 T1‐lung metastasis in breast tumor‐bearing mice (Zhao et al., 2021) and our data indicates upregulation of FoxP3 expression in the primary tumor, but not the lungs. Finally, we document a decrease in CD8 expression, a marker for anti‐tumor cytotoxic T cells, in the primary tumor. These data suggest that surgical wounding promotes a more tumorigenic immune environment in the primary tumor as well as in the lungs, albeit to a lesser extent.

We acknowledge that there are some limitations to the current study. First, only one mouse model of breast cancer was used which is not representative of all breast cancer models or patient subtypes. Additional studies using various mouse models of different breast cancer subtypes are needed to comprehensively understand the impact of surgery wounding on tumor growth and metastasis. Second, while our data indicates strong support for a role of macrophages, particularly M2 macrophages, in the surgical wounding induced promotion of lung metastasis, our study was not designed to determine causation; studies using macrophage depletion models are needed to conclusively establish a mechanistic role for macrophages. Thirdly, our immune data and subsequent interpretations are limited to one time‐point (i.e. week 5); assessment of earlier time‐points is likely to add to our knowledge of the process of immune dysregulation and subsequent metastases associated with wounding in breast cancer. Finally, our 4 T1‐luciferase2‐red fluorescent protein (modified‐4 T1), purposely used for this study given that it displays reduced lung metastatic occurrence in comparison with the rapid growth and metastasis of parental 4 T1 breast tumors (Atiya et al., 2019) may mount an immune response against luciferase and consequently limiting tumor invasiveness. While this has not been tested in our laboratory others have reported such response (Baklaushev et al., 2017), which imposes limitations to this model.

In conclusion, the current data using a TNBC model greatly contributes to the elucidation of the role of surgical wounding in accelerated breast cancer growth and metastasis. Indeed, we convincingly show that surgery wounding itself, on the contralateral side of the tumor, is sufficient to accelerate both local and distant tumor growth in a 4 T1 orthotopic model of TNBC, a response that may be mediated, at least in part, by macrophages. Future studies should examine if depletion of macrophages diminishes the tumor‐ and metastasis‐ promoting effects of surgical wounding to conclusively establish a role for macrophages. Further, given that there are no effective treatments for breast cancer metastatic relapse, future studies should assess strategies, including natural compounds, for their ability to inhibit surgical wounding‐triggered promotion of pro‐tumoral macrophage responses and consequently suppress the resulting accelerated primary breast tumor growth and lung metastasis.

### AUTHOR CONTRIBUTIONS

**Sierra J. McDonald**: Conceptualization; Investigation; Methodology; Writing – original draft; Writing – review and editing. **Brandon N. VanderVeen**: Investigation; Methodology; Writing – review and editing. **Brooke M. Bullard**: Investigation; Methodology; Writing – review and editing. **Thomas D. Cardaci**: Investigation; Methodology; Writing – review and editing. **Sarah S. Madero**: Investigation; Methodology; Writing – review and editing. **Ioulia Chatzistamou**: Investigation; Methodology; Writing – review and editing. **Daping Fan**: Conceptualization; Funding Acquisition; Methodology; Resources; Supervision; Writing – review and editing. **E. Angela Murphy**: Conceptualization; Funding Acquisition; Methodology; Resources Supervision; Writing – original draft; Writing – review and editing.

## FUNDING INFORMATION

This work was supported by NIH grants R01CA218578 and 3R01CA218578‐03S1 (to DF and EAM).
